# Clinical decision upon resection or observation of ocular surface dermoid lesions with the visual axis unaffected in pediatric patients

**DOI:** 10.1186/s40064-015-1326-7

**Published:** 2015-09-21

**Authors:** Toshihiko Matsuo

**Affiliations:** Department of Ophthalmology, Okayama University Medical School and Graduate School of Medicine, Dentistry, and Pharmaceutical Sciences, 2-5-1 Shikata-cho, Okayama City, 700-8558 Japan

**Keywords:** Limbal dermoid, Dermoid cyst, Dermolipoma, Ocular surface, Conjunctival fornix, Astigmatism, Clinical decision, Preauricular appendages or tags, Amblyopia, Surgery

## Abstract

Ocular surface or epibulbar dermoid lesions may present as limbal dermoids at the corneal limbus or dermolipomas in the conjunctival fornix. The purpose of this study is to review clinical features of ocular surface dermoids (grade I), with the visual axis unaffected, in pediatric patients, in order to find key features for making clinical decision, either resection or observation. The study involved 13 consecutive patients with limbal dermoids or fornix dermolipomas which did not affect the visual axis, seen in 11 years at a referral-based institution. Eight patients underwent surgical resection at the age, ranging from 1 to 18 (median, 4) years, with concurrent preauricular appendage resection in 3 patients. Limbal dermoids in 6 patients presented dome-shaped elevation from the ocular surface, and extended in inferotemporal quadrant for 1–2 clock hours. The remaining 2 patients showed dermolipomas in the temporal conjunctival fornix. Postoperative astigmatism at the final visit ranged from 0 to 7 (median, 2.9) diopters in 6 patients with limbal dermoids while ranged from 0 to 1 diopters in 2 patients with fornix dermolipomas. All patients with meaningful astigmatism wore glasses before and after the surgery, resulting in no apparent ametropic or anisometropic amblyopia. Observation was chosen in 5 patients with the age at initial visit, ranging from 0 to 2 (median, 1) years, and the age at the final visit, ranging from 2 to 6 (median, 3) years. Flat limbal dermoids, extending for 1–2 clock hours, were noted in 3 patients, a dome-shaped limbal dermoid for 1 clock hour in one, and a fornix dermolipoma in one. Three patients had preauricular appendages. No patient, except for one with a dome-shaped limbal dermoid, showed astigmatism, greater than one diopter. In conclusions, dome-shaped limbal dermoids were excised while flat limbal dermoids observed. The age at surgery varied largely in pediatric patients with limbal dermoids (grade I) or fornix dermolipomas which did not affect the visual axis. The surgical timing was influenced by surgical scheduling for preauricular appendage resection, determined by a plastic surgeon. Surgical decision was made for cosmetic purposes, but not for medical needs to avoid amblyopia.

## Background

Dermoid cysts (dermoids) represent the misplacement of dermal tissues, usually as globular structure, which consist of dermal stratified epithelium, subdermal connective tissue, and dermis-associated subsystems, such as hair follicles, water- and lipid-secreting glands (Pirouzian 2013). The dermal epithelium of the cysts sometimes shows keratinzation. Dermoids frequently develop around bone fissures, especially along the frontozygomatic fissure, and thus, are predominantly located inside and around the orbit of the head as ocular surface, subcutaneous, or orbital deep tissue lesions.

In ophthalmic practice, dermoids present as either ocular surface (epibulbar) lesions (Pirouzian 2013) or subcutaneous and orbital cystic lesions (Shields and Shields 2004; Cavazza et al. 2011). Periorbital subcutaneous dermoids are sometimes associated with preauricular appendages or tags. These skin lesions are often resected surgically by plastic surgeons. Therefore, the subcutaneous dermoids are not necessarily presented to ophthalmologists.

In contrast, ocular surface dermoid lesions present either as limbal dermoids at the inferotemporal corneal limbus or dermolipomas in the conjunctival fornix on the temporal side (Martinez and Cohen 1998). All these ocular surface dermoids or dermolipomas are naturally presented to ophthalmologists. In addition, the association of ocular surface dermoids or dermolipomas with preauricular appendages leads to the referral of children from plastic surgeons to ophthalmologists. Under the circumstances, a consecutive series of patients with ocular surface dermoids or dermolipomas at an eye clinic in a referral-based hospital provides a representative group of patients without bias at the presentation.

Clinical decision towards surgical intervention is made easily in pediatric patients with ocular surface dermoids or dermolipomas which affect the visual axis. In contrast, clinical decision, as for surgical intervention or observation, would be difficult to be made in pediatric patients with ocular surface dermoids or dermolipomas which do not affect the visual axis, designated as grade I. So far, no study has ever addressed this question on clinical decision-making in ocular surface dermoids or dermolipomas (grade I). In this study, 13 consecutive patients with ocular surface dermoids or dermolipomas at one institution were reviewed to describe their clinical features from the viewpoint of clinical decision, either surgical resection or observation.

## Results

Table 1 summarizes clinical features of 13 consecutive patients with limbal dermoids or conjunctival fornix dermolipomas: 8 with surgical resection and 5 with observation. No patient with either observation or surgical resection had irritating symptoms or redness of the eye, or the habit of eye-rubbing in the follow-up period.

**Table 1:** Clinical features of 13 consecutive patients with limbal dermoids or conjunctival fornix dermolipomas

Case no./sex/laterality	The age at initial visit	The age at surgery	The age at final visit	Location	Size (clock hour) and shape	Preauricular appendage	Refractive error in affected eye at earlier visit Diopter (axis)	Refractive error in affected eye/contralateral eye at final visit Diopter (axis)	Best-corrected visual acuity in affected eye/contralateral eye at final visit
Surgery									
1/M/Left	1 yr. 9 mo.	2 yr. 4 mo.	12 yr. 8 mo.	Limbus	1 h	No	s. + 1.0 c. − 1.0 (165)	s. + 1.0 c. − 1.0 (180)	1.2
					Dome-like			s. + 0.75 c.0	1.2
2/M/Left	3 mo.	1 yr. 5 mo.	7 yr. 11 mo.	Limbus	1 h	Yes	s.0 c. − 1.0 (135)	s. + 0.5 c.0	1.2
					Dome-like	Resection^a^		s. + 0.5 c.0	1.2
3/F/Right	4 yr. 0 mo.	4 yr. 8 mo.	8 yr. 8 mo.	Limbus	2 h	No	s. + 4.0 c. − 1.5 (180)	s. + 3.5 c. − 1.75 (180)	1.2
					Dome-like			s. + 2.25 c.0	1.2
4/M/Right	5 yr. 0 mo.	18 yr. 1 mo.	19 yr. 4 mo.	Limbus	2 h	No	s. + 8.5 c. − 3.5 (30)	s. + 3.0 c. − 4.0 (30)	0.7
					Dome-like			s. + 2.25 c.0	1.2
5/M/Right	2 yr. 4 mo.	4 yr. 4 mo.	9 yr. 6 mo.	Limbus	2 h	Yes	s. + 3.75 c. − 5.0 (30)	s. + 3.5 c. − 7.0 (40)	0.7
					Dome-like	Resection^a^		s. − 0.25 c. − 1.25 (90)	1.5
6/F/Left	3 yr. 7 mo.	6 yr. 5 mo.	11 yr. 0 mo.	Fornix		No	s. + 0.75 c.0	s. − 3.25 c.0	1.5
								s. − 2.5 c.0	1.5
7/F/Left	4 yr. 6 mo.	15 yr. 9 mo.	16 yr. 2 mo.	Limbus	2 h	No	s. + 2.5 c. − 4.0 (165)	s. + 0.5 c. − 5.0 (165)	1.0
					Dome-like			s. − 0.5 c. − 1.5 (165)	1.0
8/F/Right	2 mo.	1 yr. 1 mo.	2 yr. 2 mo.	Fornix		Yes	Not measured	s.0 c. − 1.0 (65)	Not measured
						Resection^a^		s.0 c. − 1.0 (75)	Not measured
No surgery									
9/M/Right	1 mo.	No surgery	3 yr. 0 mo.	Limbus	2 h	No	s. + 0.5 c.0	s. − 4.25 c. − 0.75 (60)	1.0
					Flat			s. − 3.75 c. − 0.5 (90)	1.0
10/M/Right	1 yr. 11 mo.	No surgery	6 yr. 1 mo.	Fornix		Yes	s. + 1.5 c.0	s. + 0.5 c.0	1.2
								s. + 0.5 c.0	1.2
11/F/Right	4 mo.	No surgery	3 yr. 2 mo.	Limbus	1 h	Yes	s. + 1.0 c.0	s. + 4.5 c. − 2.75 (60)	0.8
					Dome-like			s. + 0.5 c.0	0.8
12/M/Left	2 yr. 5 mo.	No surgery	3 yr. 9 mo.	Limbus	1 h	No	s. + 1.0 c. − 0.5 (180)	s. + 0.5 c.0	1.0
					Flat			s. + 0.5 c. − 1.0 (140)	1.0
13/M/Right	1 yr. 1 mo.	No surgery	2 yr. 6 mo.	Limbus	2 h	Yes	s. + 0.5 c. − 0.5 (180)	s. + 0.5 c. − 0.5 (180)	Not measured
					Flat	Resection^b^		s. + 0.5 c.0	Not measured

Refractive error at earlier visit means the error at initial visit or at the visit, nearest to the initial visit

Visual acuity in decimals, determined by Landolt-C

“Not measured” due to younger age

*M* male, *F* female, *Limbus* inferotemporal corneal limbus, *fornix* temporal conjunctival fornix, *yr.* year, *mo.* month, *h* hour, *s.* spherical lens, *c.* cylindrical lens

^a^Concurrent resection of preauricular appendage, together with dermoid

^b^Resection of preauricular appendage at the age of 1 year

The 8 patients (4 males and 4 females) underwent surgical resection at the age, ranging from 1 to 18 (median, 4) years, with concurrent preauricular appendage resection in 3 patients (Cases 2, 5, and 8). The age of the 8 patients with surgery at the initial visit ranged from 0 to 5 (median, 3) years, and the age at the final visit ranged from 2 to 19 (median, 9) years. The follow-up period from the initial visit to the final visit ranged from 2 to 14 years (median, 7 years), and the follow-up period after the surgery in the 8 patients ranged from 5 months to 10 years (median, 4.5 years). No apparent changes in the size of dermoids were noted in any patients in the follow-up before the surgical resection.

**Fig. 1:**
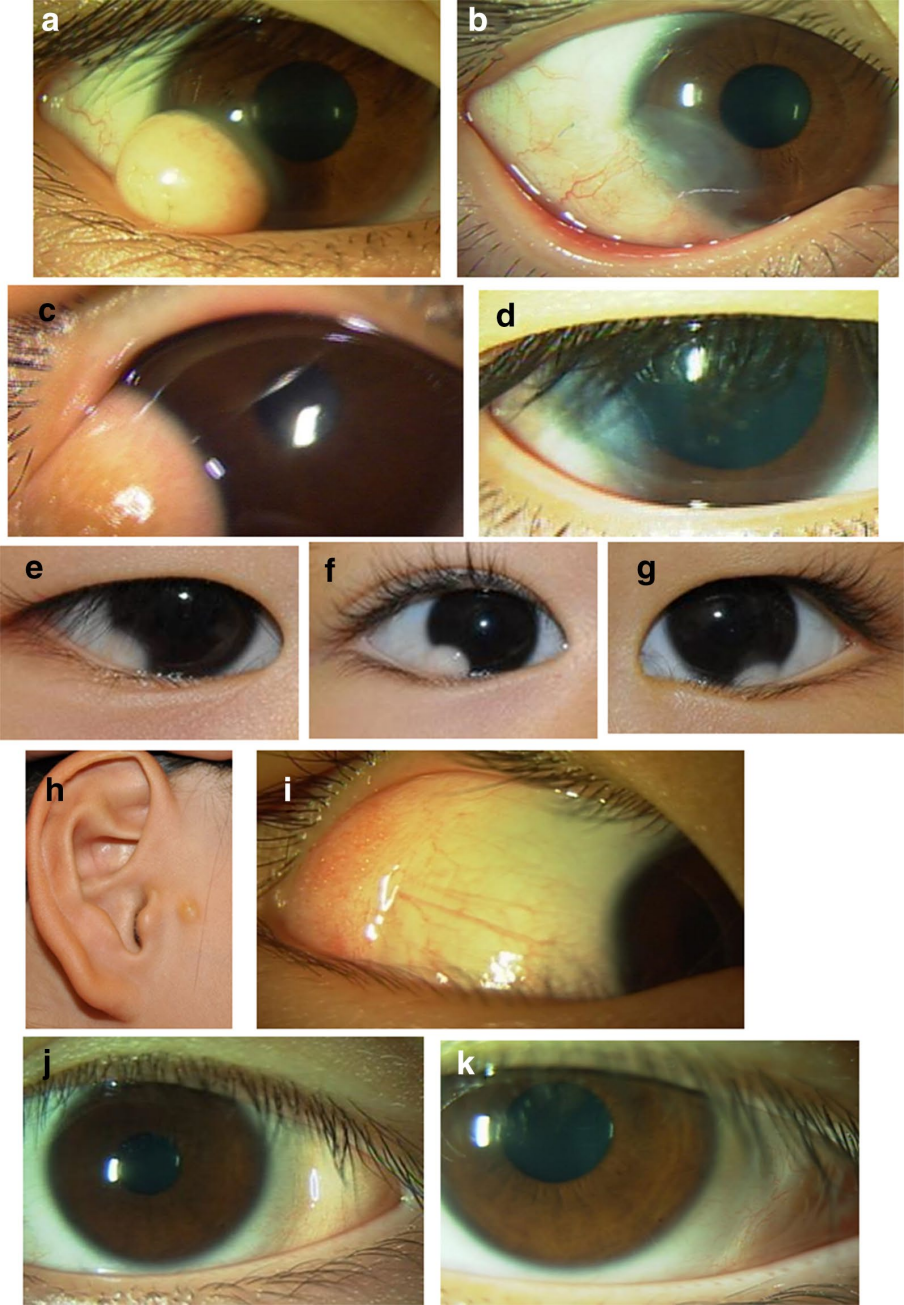
Case 4 (18-year-old male) with limbal dermoid (**a**) in the right eye, and 3 months after the resection (**b**). Case 5 (4-year-old male) with limbal dermoid (**c**) in the right eye and, 4 years after the resection (**d**). Case 9 (2-year-old male) with limbal dermoid (**e**) in the right eye. Case 11 (2-year-old female) with limbal dermoid (**f**) in the right eye. Case 12 (2-year-old male) with limbal dermoid (**g**) in the left eye. Case 10 (6-year-old male) with preauricular appendage (**h**) on the right side and temporal conjunctival fornix dermolipoma (**i**) in the right eye. Case 6 (6-year-old female) with temporal conjunctival fornix dermolipoma (**j**) in the left eye, and 3 and a half years after the resection (**k**)

Of these 8 patients with surgery, 6 patients (4 males and 2 females) had limbal dermoids with dome-shaped elevation from the ocular surface (Fig. 1a, c), extending for 1–2 clock hours in inferotemporal quadrant of the right eye in 3 patients and of the left eye in 3 patients. The remaining 2 patients (2 females) with surgery had conjunctival fornix dermolipomas on the temporal side of the right eye in one patient (Case 8) and of the left eye in one patient (Case 6, Fig. 1j).

Limbal dermoids in 6 patients were resected and the sclera was kept bare, with the conjunctiva sutured with 8-0 silk strings directly on the scleral surface as far apart as possible from the corneal limbus (Fig. 1b, d). Fornix dermolipomas in 2 patients were resected altogether with abnormal dermis-like conjunctiva, and the normal conjunctival ends were sutured with 8-0 silk strings together to the cover the sclera completely (Fig. 1k). Antibiotic and corticosteroid eye drops were discontinued at 1 month after the surgery. In the follow-up period after simple resection of limbal dermoids in 6 patients, conjunctival invasion to the resected site of the cornea beyond the limbus was at tolerable levels from the cosmetic point of view (Fig. 1b, d). All 8 patients with the surgery had no surgical complications and did not show conjunctival cicatrization or retraction in the follow-up period after the surgical resection. All 6 patients with limbal dermoids did show superficial flat corneal opacification and minimal neovascularization in the areas where the dermoids were resected. The patients and their families were satisfied with the surgical outcome.

Postoperative astigmatism at the final visit ranged from 0–7 (median, 2.9) diopters in 6 patients with limbal dermoids while ranged from 0 to 1 diopters in 2 patients with conjunctival fornix dermolipomas (Table 1). All patients underwent cycloplegic refraction with topical 1 % cyclopentolate once a year, and 4 patients with meaningful astigmatism wore full-correction glasses throughout the course before and after the surgery. The resection of limbal dermoids did not significantly influence the degree of astigmatism in the statistical comparison between the earlier visit and the final visit (*P* > 0.05, Wilcoxon rank sum test). All patients kept the best-corrected visual acuity of 0.7 or better in both eyes, and thus, no patient developed apparent ametropic or anisometropic amblyopia.

Observation was chosen in 5 patients (4 males and 1 females) with the age at the initial visit, ranging from 0 to 2 (median, 1) years, and the age at the final visit, ranging from 2 to 6 (median, 3) years. The follow-up period ranged from 1 to 4 years (median, 2 years). Three patients showed flat limbal dermoids (Fig. 1e, g), and one showed a dome-shaped limbal dermoid (Case 11, Fig. 1f), extending for 1–2 clock hours, on the inferotemporal side in the right eye of 3 patients and in the left eye of one patient. A conjunctival fornix dermolipoma on the temporal side was noted in the right eye of one patient (Case 10, Fig. 1i). Three patients had preauricular appendages (Fig. 1h). No patient, except for one with a dome-shaped limbal dermoid (Case 11, Fig. 1f), showed astigmatism, greater than one diopter, on cycloplegic refraction. No apparent changes in the size of dermoids were noted in the follow-up of any patients with observation.

## Discussion

Ocular surface dermoids or dermolipomas are one kind of representative diseases in pediatric ophthalmology, together with congenital cataract, congenital blepharoptosis, and ectopia lentis (Matsuo 2014, 2015). Pediatric eye diseases, in general, have standpoints which are different from those in adult eye diseases. Firstly, the eyeball is in the process of growth and the visual acuity is in the process of development. Secondly, general anesthesia is required in pediatric eye surgeries. Under the circumstances, the timing for surgical intervention has to be determined along the line of avoiding and treating amblyopia in pediatric eye diseases.

Ocular surface dermoids, which affects the visual axis, would be resected under medically needed guidelines rather than for cosmetic purposes, as described above. In contrast, surgical indication remains unclear in ocular surface dermoids or dermolipomas which do not affect the visual axis. The goal of this study is set, therefore, to review clinical decision-making, regarding intervention or observation, in the consecutive series of patients with ocular surface dermoids or dermolipomas which did not affect the visual axis.

A common clinical feature of limbal dermoids in 6 patients who underwent surgical resection was dome-shaped appearance, involving 1–2 clock hours along the inferotemporal limbus. In contrast, limbal dermoids with observation were flat in shape, involving 1–2 clock hours at the inferotemporal limbus, in all 4 children except for one with dome-shaped appearance. The cosmetic problem, regarding the dome-shaped appearance of limbal dermoids, naturally led patients and their families to the choice of surgical intervention. It should be noted that the surgical timing for resection of limbal dermoids largely varied from patient to patient. The determination of surgical timing would be dependent, not only on the family’s way of thinking, but also on doctors’ attitude, as mentioned below.

The association of preauricular appendage or tags with ocular surface dermoids or dermolipomas in children did influence the surgical timing: ocular surface lesion resection by an ophthalmologist and preauricular appendage resection by a plastic surgeon are usually scheduled at the same time under a single session of general anesthesia. Family members often wished for concurrent resection of an ocular surface dermoid lesion in a child on the scheduled date of general anesthesia when the preauricular appendage resection would be planned by a plastic surgeon. The referral and consultation between an eye doctor and a plastic surgeon would lead to the fact that children with preauricular appendages underwent resection of ocular surface dermoids or dermolipomas at the earlier age in this study.

Large dome-shaped limbal dermoids were naturally associated with large degree of astigmatism. Indeed, in this study (Table 1), astigmatism was trivial in dome-shaped dermoids, extending only for 1 clock hour (Cases 1 and 2) while meaningful astigmatism was present in dome-shaped dermoids, extending for 2 clock hours (Cases 3, 4, 5, and 7). Anisometropic or ametropic amblyopia would develop if such astigmatism is left behind without appropriate correction by glasses. It should be noted that the degree of astigmatism did not significantly change in the course of follow-up, before and after the surgery. The persistent astigmatism after the resection of limbal dermoids has been also described in a preceding study (Robb 1996). Since the ocular surface dermoid lesions have not enlarged in size, and hence, the degree of astigmatism has not changed in the follow-up, the presence of anisometropic astigmatism or non-compliance of children to spectacle wear could not be used as a clinical guidance for surgical resection.

Taking a closer look at the change in astigmatism (Table 1), two children (Cases 5 and 7) who underwent limbal dermoid resection did show the astigmatic increase by one to two diopters between the earlier visit and the final visit. In addition, one child (Case 11) with a dome-shaped limbal dermoid has developed about 2-diopter astigmatism in the period of observation. The astigmatic increase would be attributed to the normal development of the cornea in the process of growth.

The visual acuity check-up is recommended in all children with ocular surface dermoids or dermolipomas, irrespective of wearing full-correction glasses which are prescribed by cycloplegic refraction once a year. It should be noted in this study that two children (Cases 4 and 5) showed meaningful reduction of best-corrected visual acuity in the eyes with limbal dermoid resection at the final visit, compared to the contralateral eyes (Table 1), indicating the presence of mild anisometropic amblyopia. The visual acuity in the involved eyes in these two children was 0.7 which would be considered as an acceptable level for daily life. Under the circumstances, patching (occlusion therapy) on the contralateral eyes was not adopted in any children in this study.

To the best of my knowledge, this study is the first to address a question upon clinical decision-making, either surgical intervention or observation, in pediatric patients with ocular surface dermoids or dermolipomas which do not affect the visual axis. The patients with dome-shaped limbal dermoids chose surgical resection while the patients with flat-shaped dermoids chose observation. Surgical timing for ocular surface dermoid or dermolipoma resection by an ophthalmologist was influenced by the consultation with a plastic surgeon who was planning preauricular appendage resection. Large dome-shaped limbal dermoids were associated with large degree of astigmatism while flat limbal dermoids in observation and conjunctival fornix dermolipomas were not associated with astigmatism. Full correction of astigmatism with glasses was mandatory to avoid anisometropic and ametropic amblyopia from the earlier age of patients with dome-shaped limbal dermoids.

A recommendation, based on this clinical series, is that ocular surface dermoids or dermolipomas, not involving the visual axis, and hence, not impairing the visual acuity, would be observed for a period of time at first. In the case that parents are concerned with cosmetic appearance of the lesions in children, surgical resection would be recommended as a clinical option in addition to observation. Surgical timing is basically left to the discretion of the families. A risk of general anesthesia, even though at a low level, must be considered, especially in surgeries for cosmetic purposes. For instance, a surgery would be scheduled concurrently with plastic surgeons, removing the preauricular appendages. Avoiding and treating amblyopia cannot be used as a clinical guidance for making the decision of either resection or observation in the ocular surface dermoids or dermolipomas which do not affect the visual axis. Imaging with anterior-segment ultrasound biomicroscopy or optical coherence tomography would assist in making surgical decision and conducting surgical procedures in safety.

## Conclusions

Dome-shaped limbal dermoids were excised while flat limbal dermoids observed. The age at surgery varied largely in pediatric patients with limbal dermoids (grade I) or fornix dermolipomas which did not affect the visual axis. The timing of surgical intervention was also influenced by surgical scheduling for preauricular appendage resection, determined by a plastic surgeon. The surgical decision would be, therefore, based on the following situations: (1) limbal dermoids appear as dome-shaped, (2) the other pending scheduled surgeries, such as preauricular tag removal, are concurrently considered, (3) the parents wish for the surgical removal, and (4) the patients have injection of the lesions and hence, symptomatic irritation which leads to the habit of eye rubbing.

## Methods

Retrospective review was made on medical records of 13 consecutive patients with ocular surface dermoid lesions, including limbal dermoids and conjunctival fornix dermolipomas, who were seen at Okayama University Hospital in 11 years until December 2014. In the study period, there was no patient with ocular surface dermoids which affected the visual axis. The study, therefore, set neither inclusion criteria nor exclusion criteria. All surgeries were done by a single surgeon (T. M.) under general anesthesia, except for two patients (Cases 4 and 7) who underwent surgery under local anesthesia at the age of 18 and 15 years, respectively. All resected lesions, including limbal dermoids and conjunctival fornix dermolipomas, were submitted to the pathology laboratory of the hospital and confirmed histopathologically as dermoids and dermolipomas, respectively. The study adhered to the tenets of Declaration of Helsinki and was approved as a retrospective case-series study by Ethics Committee of Okayama University Graduate School of Medicine, Dentistry, and Pharmaceutical Sciences.

The data, collected from medical records, were limbal dermoids or fornix dermolipoma, dome-shaped or flat configuration in limbal dermoids, their clock-hour extension along the corneoscleral limbus as the size of the lesions, the concurrent presence of preauricular appendages, the sex, the age at the initial visit and final visit, the age at surgery, refractive errors and best-corrected visual acuity in both eyes, the presence or the absence of symptomatic irritation, parents’ satisfaction with surgical outcome, and surgical or postoperative complications. The lesions were not measured with anterior-segment ultrasound biomicroscopy or optical coherence tomography. Orbital magnetic resonance imaging or computed tomographic scan was not performed.

All patients were followed every 4 months for vision care. The visual acuity at distant viewing was tested at the distance of 5 m with internationally standard Landolt-C charts. Children at the younger age were tested with Landolt-C cards at 2.5 m for distant viewing and the visual acuity at 2.5 m was converted to that presumed at the measuring distance of 5 m. The visual acuity at near viewing was tested at about 30 cm with a hand-held Landolt-C cards for near viewing. The intraocular pressure in children was measured with a hand-held tonometer (Icare TA01i, Icare Finland, Helsinki). The other standard ophthalmological examinations included hand-held or table-fixed autorefraction, hand-held slit-lamp biomicroscopy, and funduscopy (Matsuo 2014).
